# Supplementary material for: Heart disease in a mutant mouse model of spontaneous eosinophilic myocarditis maps to three loci
Source: BMC Genomics. 2019 Oct 11;20:727. doi: 10.1186/s12864-019-6108-0 (PMC6788080; doi:10.1186/s12864-019-6108-0)
Supplement: Supplementary file 1 — Additional file 1. File contains a figure that shows an overview and outcomes from 4 rounds of breeding crosses to map the EM/HD trait. [file 12864_2019_6108_MOESM1_ESM.pptx]

## Slide 1
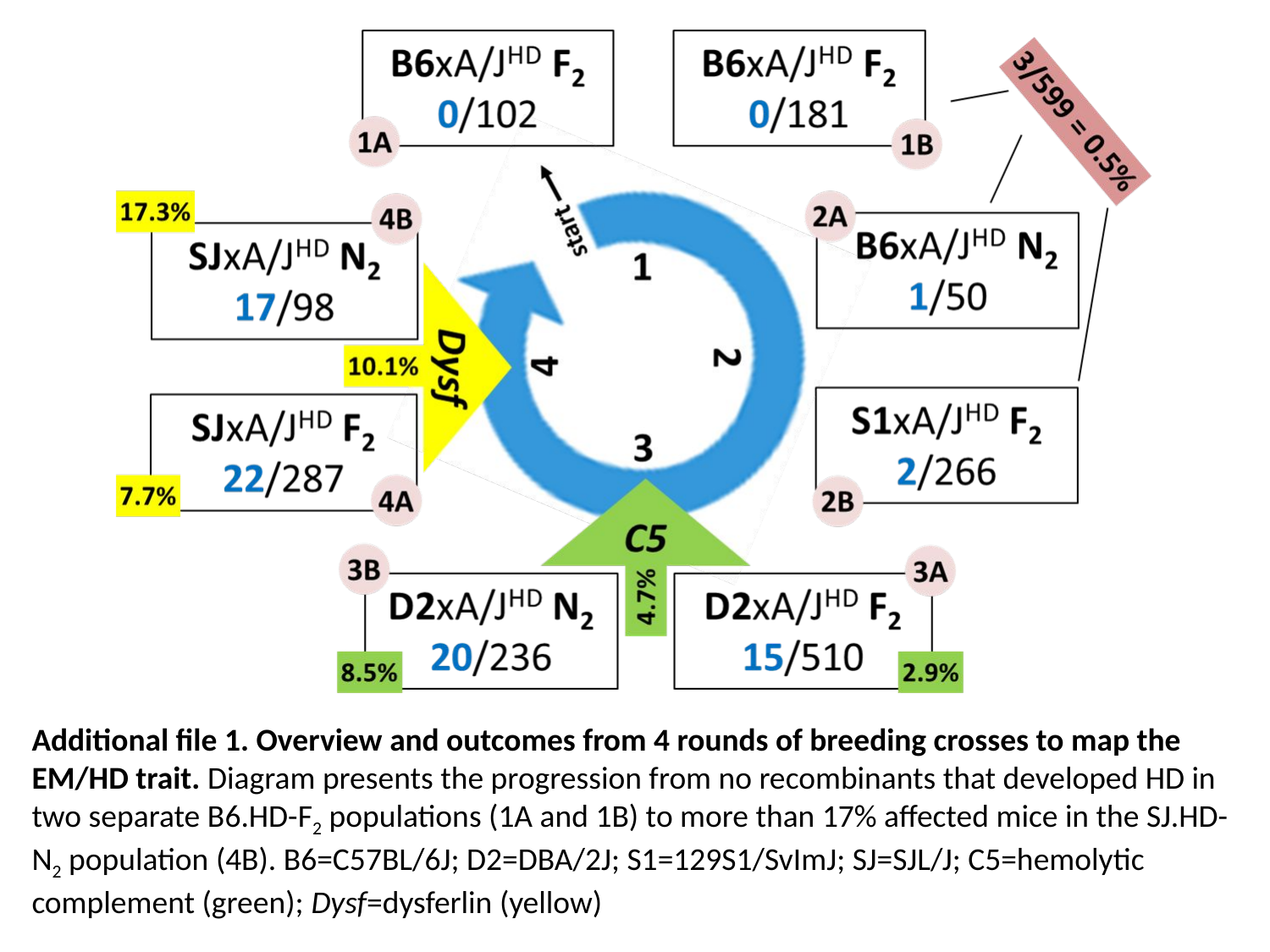

Additional file 1. Overview and outcomes from 4 rounds of breeding crosses to map the EM/HD trait. Diagram presents the progression from no recombinants that developed HD in two separate B6.HD-F2 populations (1A and 1B) to more than 17% affected mice in the SJ.HD-N2 population (4B). B6=C57BL/6J; D2=DBA/2J; S1=129S1/SvImJ; SJ=SJL/J; C5=hemolytic complement (green); Dysf=dysferlin (yellow)
