# Supplementary material for: Heart disease in a mutant mouse model of spontaneous eosinophilic myocarditis maps to three loci
Source: BMC Genomics. 2019 Oct 11;20:727. doi: 10.1186/s12864-019-6108-0 (PMC6788080; doi:10.1186/s12864-019-6108-0)
Supplement: Supplementary file 2 — Additional file 2. File contains the QTL analysis results of D2.HD-F2 and the combined D2.HD-N2 and D2.HD-F2 cohorts. [file 12864_2019_6108_MOESM2_ESM.pptx]

## Slide 1
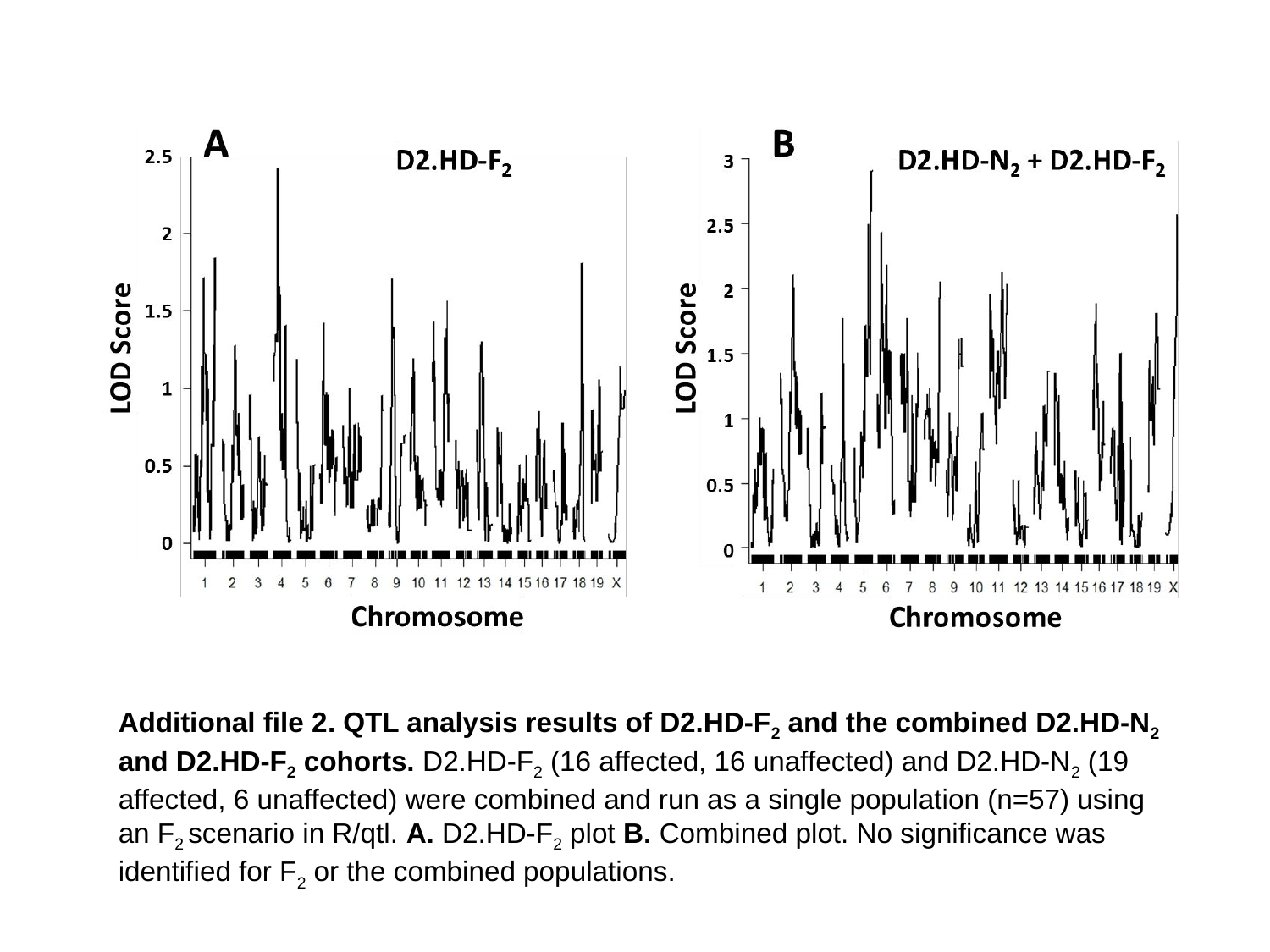

Additional file 2. QTL analysis results of D2.HD-F2 and the combined D2.HD-N2 and D2.HD-F2 cohorts. D2.HD-F2 (16 affected, 16 unaffected) and D2.HD-N2 (19 affected, 6 unaffected) were combined and run as a single population (n=57) using an F2 scenario in R/qtl. A. D2.HD-F2 plot B. Combined plot. No significance was identified for F2 or the combined populations.
