# Supplementary material for: Heart disease in a mutant mouse model of spontaneous eosinophilic myocarditis maps to three loci
Source: BMC Genomics. 2019 Oct 11;20:727. doi: 10.1186/s12864-019-6108-0 (PMC6788080; doi:10.1186/s12864-019-6108-0)
Supplement: Supplementary file 3 — Additional file 3. File contains the QTL analysis results of the combined SJ.HD-N2 and SJ.HD-F2 cohorts. [file 12864_2019_6108_MOESM3_ESM.pptx]

## Slide 1
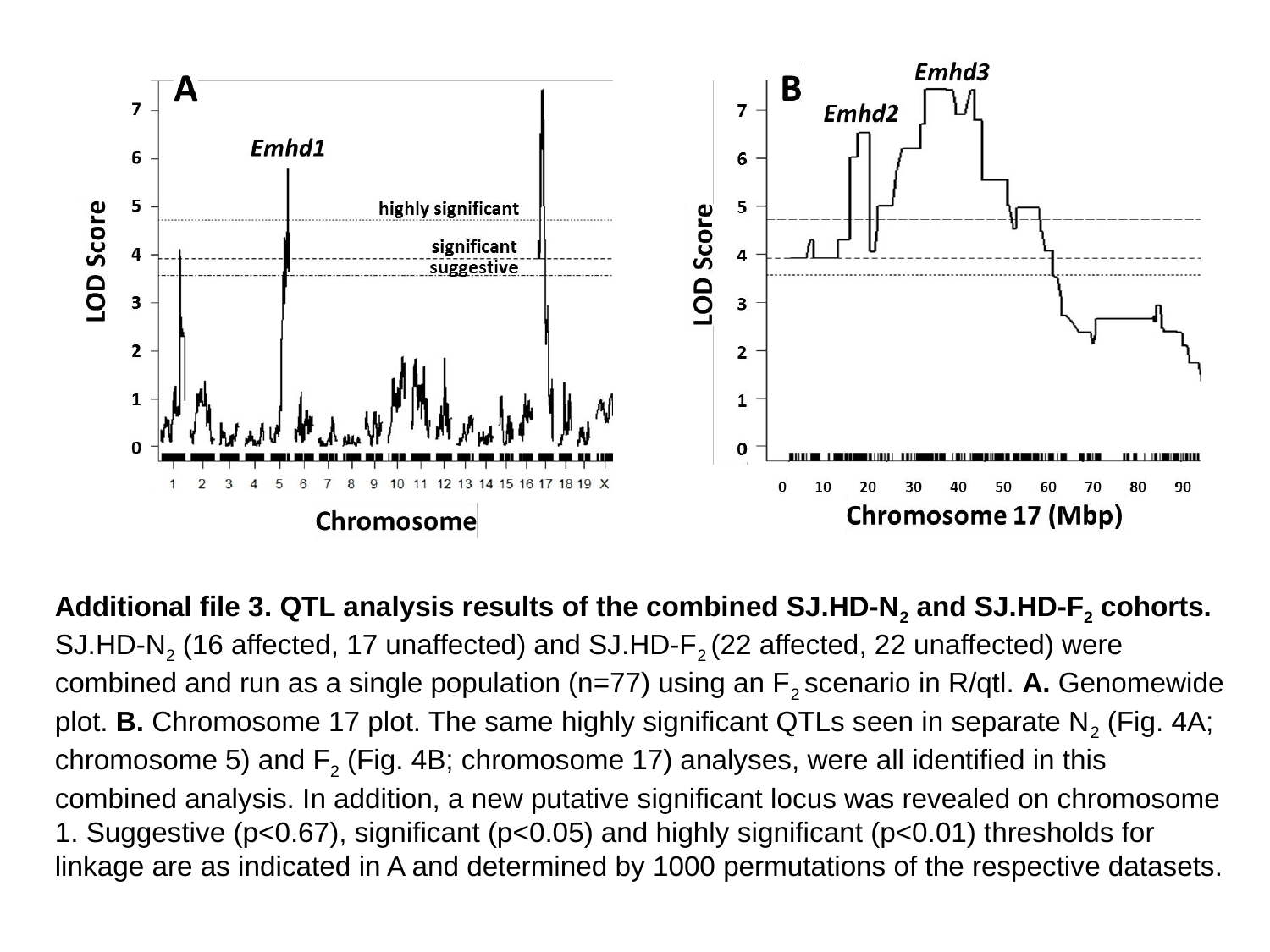

Additional file 3. QTL analysis results of the combined SJ.HD-N2 and SJ.HD-F2 cohorts. SJ.HD-N2 (16 affected, 17 unaffected) and SJ.HD-F2 (22 affected, 22 unaffected) were combined and run as a single population (n=77) using an F2 scenario in R/qtl. A. Genomewide plot. B. Chromosome 17 plot. The same highly significant QTLs seen in separate N2 (Fig. 4A; chromosome 5) and F2 (Fig. 4B; chromosome 17) analyses, were all identified in this combined analysis. In addition, a new putative significant locus was revealed on chromosome 1. Suggestive (p<0.67), significant (p<0.05) and highly significant (p<0.01) thresholds for linkage are as indicated in A and determined by 1000 permutations of the respective datasets.
