# Supplementary material for: Heart disease in a mutant mouse model of spontaneous eosinophilic myocarditis maps to three loci
Source: BMC Genomics. 2019 Oct 11;20:727. doi: 10.1186/s12864-019-6108-0 (PMC6788080; doi:10.1186/s12864-019-6108-0)
Supplement: Supplementary file 7 — Additional file 7. File contains a figure summarizing the breeding schemes for mapping the HD trait. [file 12864_2019_6108_MOESM7_ESM.pptx]

## Slide 1
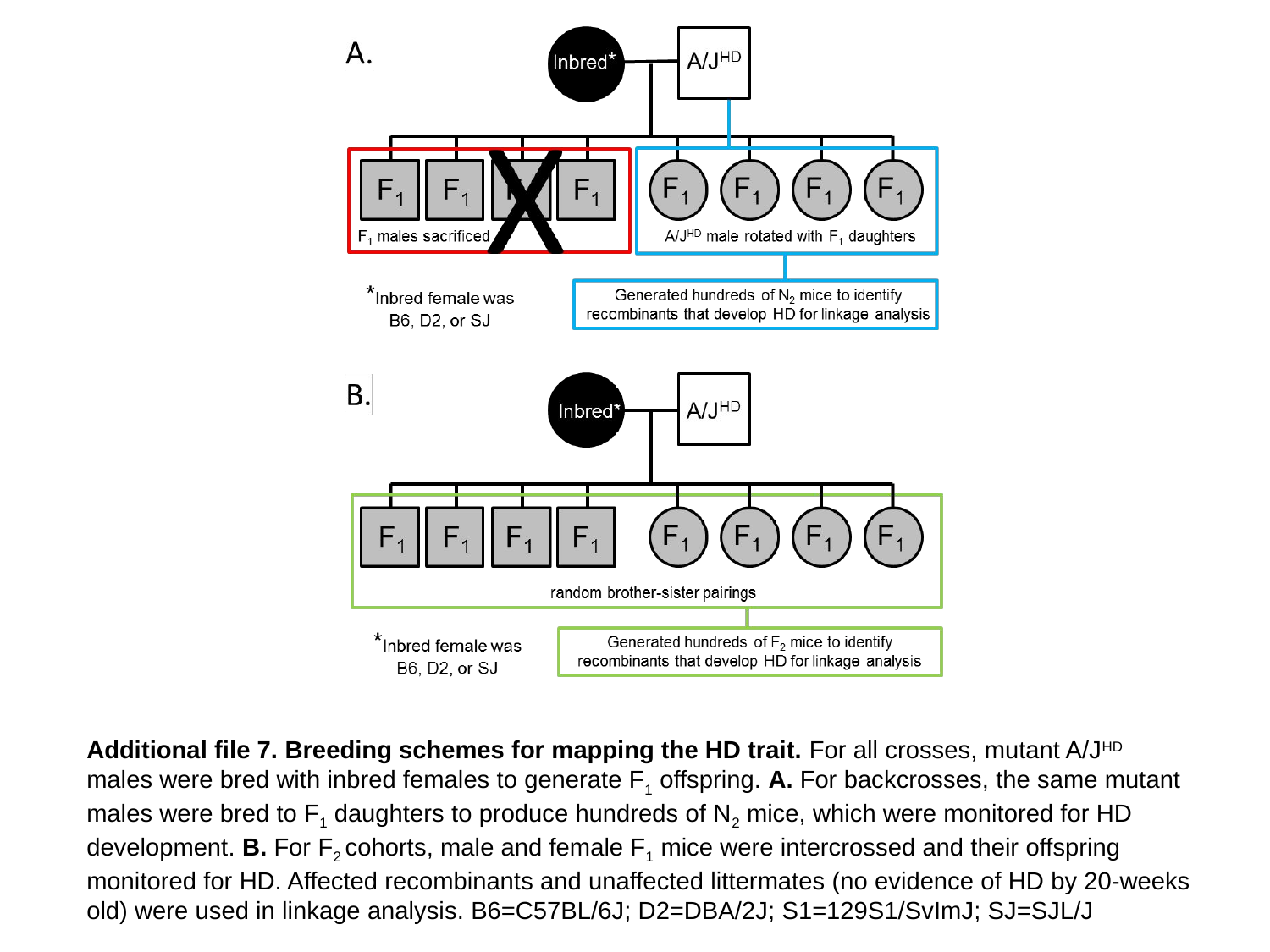

Additional file 7. Breeding schemes for mapping the HD trait. For all crosses, mutant A/JHD males were bred with inbred females to generate F1 offspring. A. For backcrosses, the same mutant males were bred to F1 daughters to produce hundreds of N2 mice, which were monitored for HD development. B. For F2 cohorts, male and female F1 mice were intercrossed and their offspring monitored for HD. Affected recombinants and unaffected littermates (no evidence of HD by 20-weeks old) were used in linkage analysis. B6=C57BL/6J; D2=DBA/2J; S1=129S1/SvImJ; SJ=SJL/J
